# Updated IQ and Well-Being Scores for the 50 U.S. States

**DOI:** 10.3390/jintelligence10010015

**Published:** 2022-02-27

**Authors:** Bryan J. Pesta

**Affiliations:** Department of Management, Cleveland State University, Cleveland, OH 44115, USA; b.pesta@csuohio.edu

**Keywords:** state IQ, aggregate IQ, well-being, g nexus, PIAAC, NAEP

## Abstract

At the level of the 50 U.S. states, an interconnected nexus of well-being variables exists. These variables strongly correlate with estimates of state IQ in interesting ways. However, the state IQ estimates are now more than 16 years old, and the state well-being estimates are over 12 years old. Updated state IQ and well-being estimates are therefore needed. Thus, I first created new state IQ estimates by analyzing scores from both the Program for the International Assessment of Adult Competency (for adults), and the National Assessment of Educational Progress (for fourth and eighth grade children) exams. I also created new global well-being scores by analyzing state variables from the following four well-being subdomains: crime, income, health, and education. When validating the nexus, several interesting correlations existed among the variables. For example, state IQ most strongly predicted FICO credit scores, alcohol consumption (directly), income inequality, and state temperature. Interestingly, state IQ derived here also correlated 0.58 with state IQ estimates from over 100 years ago. Global well-being likewise correlated with many old and new variables in the nexus, including a correlation of 0.80 with IQ. In sum, at the level of the U.S. state, a nexus of important, strongly correlated variables exists. These variables comprise well-being, and state IQ is a central node in this network.

## 1. Introduction

McDaniel (2006) created aggregate-level IQ estimates for the 50 U.S. states. He did so by analyzing National Assessment of Educational Progress (NAEP) achievement scores (mathematics and reading) from fourth and eighth graders in each state. Much research has resulted from McDaniel’s (2006) estimates. Some of the state variables that IQ correlates with include government effectiveness (McDaniel 2006), crime, education, health, and income (Pesta et al. 2010), UV radiation (Leon and Hassall 2017), climate (Ryan et al. 2010), U.S. presidential election results (Pesta and McDaniel 2014), religiosity (Reeve 2009), and global well-being (Pesta et al. 2010).

Well-being is often conceptualized as one’s degree of physical and psychological health (Warr 1987, 2007). The former refers to both the absence of disease and to whether one’s basic needs are being met. The latter comprises five facets: affective well-being (i.e., affect), competence (the ability to deal with life’s demands), autonomy (the ability to resist environmental influences), aspiration (motivation toward reaching goals), and integrated functioning (the balancing of life demands).

Pesta et al. (2010) created an index of well-being for the 50 U.S. states by first coding a variety of state-level data, mostly from governmental sources (for a recent, similar approach, see Montez et al. 2020). The authors then derived first principal components by analyzing variables for the following “subdomains” of well-being: crime, income, education, health, and religiosity. These five variables, together with state IQ, were next entered into a hierarchical PCA to create a global measure of well-being. The well-being scores correlated strongly with several other important state-level variables. The correlations further supported the idea that just a single component of well-being is enough to capture most of the variance on diverse sets of state-level variables. Examples of correlations from Pesta et al. (2010) include well-being and: traffic fatalities (−0.71), the number of active doctors (0.52), the percent of Protestants (−0.68), and the percent of state residents voting for Barack Obama in the 2008 presidential election (0.47).

However, why should one worry about updating state IQ and well-being estimates? First, state IQ estimates possess impressive predictive validity across a range of outcome variables that together comprise well-being. As standardized IQ tests are updated regularly across time, it is likely that state (and national) IQ estimates need updating as well. Second, U.S. state level estimates show the same patterns as seen with national IQ estimates. Hence, each can be seen as an important replication of the other. Third, the IQ estimates have also been useful for tests of theory. Examples include Pesta and Poznanski (2014) using the estimates as a test of Cold Winters Theory, and Leon and Hassall (2017) using the estimates to test their UV Radiation model. 

In sum, both state IQ and state well-being show strong convergent validity in that they correlate with many other important, state-level outcome variables. A problem with these estimates, however, is that they are old. McDaniel’s (2006) IQ scores are 16 years old, whereas Pesta et al.’s (2010) well-being estimates are 12 years old. Therefore, my goals were to update and expand upon the older IQ and well-being scores and to attempt further mapping of the g/well-being nexus.

To achieve this, I first updated state IQ values by analyzing scores on two state-level exams: the Program for the International Assessment of Adult Competencies (PIAAC, for adults), and the National Assessment of Educational Progress (NAEP, for fourth and eighth grade children). Next, I coded current data regarding state crime, income, education, and health. These four variables served as subdomains of well-being. I then subjected scores on these variables to a hierarchical PCA, thereby deriving a global well-being measure.

After deriving state IQ and well-being, I correlated these estimates with various other “variables of interest”. These were either variables that have already been featured in the state-level IQ literature (e.g., temperature and conservatism), or variables having relevance to the United States today (e.g., COVID-19 vaccination rates and the 2020 presidential election results). My goal was to test the breadth of the g/well-being nexus by focusing on convergent validity. That is, what other important variables do state-level IQ and well-being predict?

## 2. Materials and Methods

### 2.1. Sample and Scale Construction

The unit of analyses was the 50 U.S. states. I first derived state IQ scores from PIAAC Literacy and Numeracy scores and NAEP Math and Reading scores, as described more fully below. Thereafter, I compiled data from various online sources, mostly governmental, related to either state crime, education, health, or income. These four areas comprised the subdomains of well-being. Note that each well-being subdomain was derived from multiple measures of the same construct. For example, in all, six state-level health variables were used to create the health subdomain.

I used principal components analyses to build scales for the four well-being subdomains. After this, I conducted a PCA on the four well-being principal components. As an example, I coded four “marker variables” for state-level income: household income, home values, the employment rate, and poverty. I then subjected these four marker variables to a PCA which produced a single PC explaining 74% percent of the variance in the four marker variables. Loadings ranged from 0.75 (home values) to 0.94 (household income). I carried out similar analyses with the three other well-being subdomains—health, crime, and education. Thereafter, I entered all four well-being subdomain scores (income, health, crime, and education) into a “hierarchical PCA” to derive the single, global well-being component. The aim was to test whether a single component of global well-being could best explain all the state data, or whether multiple components were needed.

### 2.2. Measures

#### 2.2.1. IQ

I derived state-level IQ partly from analyses of PIAAC Literacy and Numeracy scores (2012–2017). The PIAAC is administered to adult participants and is “an international assessment covering a broad range of abilities, from simple reading to complex problem-solving skills (PIAAC 2021).” According to the PIAAC (2021), the literacy test evaluates “the ability to understand, evaluate, use and engage with written texts to participate in society,” while the numeracy test evaluates “the ability to access, interpret, use, and communicate mathematical information to deal with the demands of a range of situations in adult life.” Additionally, the PIAAC claims its exams are “authentic, culturally appropriate, and drawn from real-life situations that are expected to be of importance or relevance in different contexts (PIAAC 2021).” Finally, PIAAC exams are highly *g-*loaded (Ganzach and Patel 2018; Gottfredson 1997).

I also derived state IQ partly from NAEP Reading and Math scores (in 2015, 2017, and 2019). This was the exam McDaniel (2006) used for his original IQ estimates as well. The NAEP is a “congressionally mandated large-scale assessment administered by the National Center for Education Statistics (NCES). It consists of assessments in [mathematics and reading] (NCES 2021).” The NCES administers the exam to fourth and eighth grade students across the USA. Moreover, because NCES administers the same assessment in every state, NAEP provides “a common measure for student achievement in public schools across the country (NCES 2021).”

Regarding what the NAEP measures, Rindermann and Thompson (2013) noted: “Both NAEP scales together measure a mixture of general intelligence and specific knowledge, covered by the construct cognitive ability… However, compared to figural scales as the Ravens, NAEP scales are more measures of crystallized knowledge.” 

Regarding reliability, I calculated test–retest correlations on year over year scores within subject areas (e.g., I analyzed NAEP math and reading scores separately for the years 2015, 2107, and 2019) for both NAEP and PIAAC. For the former, reliabilities were 0.84 (math) and 0.86 (reading). Reliabilities for year over year PIAAC scores (i.e., for numeracy and literacy) were both near unity (0.99). Thus, the reliability of the state IQ estimates here is very high, which is consistent with what McDaniel (2006) found with his original IQ estimates. 

Finally, state IQ scores were derived by taking the mean of the derived PIAAC and NAEP IQ scores.

#### 2.2.2. Crime

Crime variables were coded from either the FBI (2019) or the U.S. Bureau of Justice Statistics (2019). The first variable I coded captured property crimes (i.e., burglary, larceny-theft, motor vehicle theft, and arson), and the second captured violent crimes (i.e., murder and non-negligent manslaughter, forcible rape, robbery, and aggravated assault.). All values were ranks and came from the FBI (2019). Additionally, I coded the number of inmates per capita by U.S. state (U.S. Bureau of Justice Statistics 2019).

#### 2.2.3. Education

The first education variable I coded was the percent of state residents with at least a bachelor’s degree. These data came from the U.S. Census (2019). Next, I coded the amount of money states spend on education on a per-student basis. These values also came from the U.S. Census (2019). Third, WalletHub (2021a) “compared all 50 states across 18 metrics that examined the key factors of a well-educated population: educational attainment, school quality and achievement gaps between genders and races” (WalletHub 2021b). I coded these values as ranks. Finally, I coded U.S. News and World Reports state rankings based on educational quality (U.S. News 2021). Factors going into these rankings included pre-K enrollment, standardized test scores, and high school graduation rates.

#### 2.2.4. Health

I coded six variables to measure state health. The first was rates per 100,000 of chronic obstructive pulmonary disease. The remaining five variables were percentages. These included the percent of state residents who: are obese (BMI ≥ 30), have diabetes, have heart disease, have cognitive difficulties, or have ambulatory difficulties. All variables came from the Behavioral Risk Factor Surveillance System (BRFSS 2021). The BRFSS is “the nation’s premier system of health-related telephone surveys that collect state data about U.S. residents regarding their health-related risk behaviors, chronic health conditions, and use of preventive services (BRFSS 2021).”

#### 2.2.5. Income

All four income values came from the U.S. Census (2019). Variables included the state employment rate (%), poverty rate (%), household income (USD), and median home values (USD).

#### 2.2.6. Additional Measures

After deriving state IQ and well-being scores, I sought to identify other, interesting, or important state-level variables that might be covariates. I selected 11 such variables mostly because the state/aggregate IQ literature has focused on them. Some variables though (e.g., COVID-19 vaccination rates) were selected based on currency and author interest. At any rate, the 11 variables included state: (1). Religiosity. The percent of adults who are “highly religious” (Pew Research Center 2019). (2). Income inequality. The extent to which income is distributed unevenly among states (World Population Review 2021a). (3). Conservatism. The self-identified political ideology of residents by state (Gallup 2019). (4). COVID-19 vaccination rates. The percent of at least partially vaccinated state residents (Kaiser Family Foundation 2021; coded on 11 November 2021).

(5). The price of a pack of cigarettes in each state (World Population Review 2021b). (6). Alcohol consumption. Adults who have had at least one drink of alcohol within the past 30 days (i.e., the “Crude Prevalence”; BRFSS 2021). (7). Percent Biden. The percent of state residents voting for Joe Biden in the 2020 U.S. presidential election (CNN 2021). (8). Minimum wage. Each state’s minimum wage (PayCor 2021). For states that have not passed minimum wage laws, I used the federal minimum (USD 7.25) as values. (9). Temperature. Average temperature by state (CurrentResults 2021). (10). Gunowners. The percent of state residents owning guns (World Population Review 2021b). (11). Average FICO credit scores by state (Experian 2019).

Next, I coded the percent of White, Black, and Hispanic individuals in each state (U.S. Census 2019). These variables were used to test for suppression effects when predicting the 2020 U.S. presidential election (e.g., see, Pesta and McDaniel 2014). Conclusions reported here, however, were unchanged across these three racial/ethnic groups, and so I only report the suppression analyses using percent White below.

Lastly, I coded extant measures of U.S. state IQ. These included estimates by McDaniel (2006), and by Fuerst and Kirkegaard (2016). Interestingly, Alexander (1922) reported state IQ estimates using the Army Alpha that are now over 100 years old. I also correlated current state IQ estimates with these much older values. 

## 3. Results

I first conducted separate PCAs on the four well-being subdomains, as shown in Table 1. Note that the percent of variance explained by the first principal component was very large in each analysis. These values ranged from 75% for global well-being to 94% for income.

Table 1 also shows a correlation matrix for all principal components (plus state IQ). The correlations were consistently large, which allowed the derivation of a general component of state well-being (i.e., with the crime, education, health, and income subdomains). The general component explained 75% of the variance in the well-being subdomains. Note also that state IQ correlated strongly with all four subdomains, and 0.80 with the global well-being composite.

Table 2 shows rankings and standard scores for the 50 U.S. states by IQ, global well-being, and the well-being subdomains. The ranks are strongly consistent across IQ and all the well-being variables. For example, Massachusetts ranks second in IQ and income, and first in education and global well-being. It also ranks eighth in crime, and seventh in health. Conversely, Louisiana ranks 49th in IQ, 43rd in health, 45th in education, 46th in global well-being, 48th in income, and last in crime. 

### 3.1. State IQ

Table 3 shows a network of inter-correlated variables, including both state IQ and state well-being. Considering IQ first, this variable correlated significantly with eight of the eleven “other” variables in the table. For example, IQ correlated −0.57 with religiosity (higher religiosity translates to lower state IQ). This replicates findings from Pesta et al. (2010; see also Zuckerman et al. 2019, for similar results with individual-level data), who even included religiosity as a global well-being subdomain.

State IQ correlated inversely (−0.34) with conservatism. Several state-level articles exist on liberalism/conservatism and IQ (Meisenberg 2015; Stankov 2009). They show that state-level conservatism is associated with lower IQ. Consistent with this, state IQ also correlates strongly with state-level income inequality. That is, as IQ scores go up, state income inequality goes down (−0.51).

Next, state IQ correlates 0.38 with the percent of state residents who have been at least partially vaccinated against the COVID 19 virus. It is remarkable that state IQ correlates moderately strongly with something like COVID-19 vaccination rates. This effect may be due to links between IQ, vaccination rates, and blue versus red leaning states, as can be seen from the intercorrelations in Table 3 (e.g., vaccination rates correlate very strongly, *r* = −0.83, with state conservatism).

State IQ correlates strongly with alcohol consumption. Surprisingly, however, the relation is direct. Higher IQ is associated with higher levels of alcohol consumption (0.57). Pesta et al. (2010) first reported this effect for aggregate-level data, though Belasen and Hafer (2013) reported the same using individual-level data. Thus, there appears to be a consistent, positive correlation between IQ and alcohol consumption, and even alcohol consumption and various chronic health conditions (Pesta et al. 2012). Consider the correlations between alcohol consumption and the variables I used here to measure state health. These ranged from −0.40 (cognitive difficulties) to −0.61 (diabetes). At present, I have no explanation for why alcohol consumption is associated with better health outcomes at the state level.

Next, the percent of state residents voting for Joe Biden is only weakly correlated with IQ (0.18). However, several studies have shown that state-level suppression effects exist when trying to predict presidential election outcomes from state IQ (Pesta 2017). The pattern is that after controlling for state racial composition (e.g., percent White), higher IQ is associated with more votes cast for the democrat (and vice versa). Here, for example, state IQ correlates only 0.18. with percent Biden. After controlling for percent White (via regression), the correlation increases to 0.47. The suppression effects are mutual in that the univariate correlation between percent White and percent Biden is −0.39. When controlling for state IQ, this value becomes −0.61.

Additionally, in Table 3, state temperature correlates −0.62 with state IQ. In general, the further north the state, the higher its IQ (and well-being). This replicates previous state-level results (Pesta and Poznanski 2014), though it is inconsistent with county-level results (Pesta et al. 2020). The largest correlation in Table 3, however, is between IQ and FICO credit scores (0.87; see also the 0.85 correlation between well-being and FICO scores). The strong correlations here are perhaps not surprising given all the factors that go into a credit score. To wit, credit scores correlate significantly with every other variable in the table. Correlations range from −0.34 (percent gun owners) to 0.87 (state IQ).

Next, a reviewer wondered whether the correlations here might differ when looking at PIAAC and NAEP scores separately (versus averaged together, as in Table 3). The results were mostly trivially different across exams. For example, here are some correlations between PIAAC (or NAEP) and: conservatism, −0.30 (−0.32), vaccination rates, −0.39 (0.31), alcohol consumption, 0.50 (0.54; an exam for children strongly predicts state alcohol consumption), FICO scores 0.70 (0.88; an exam for children strongly predicts state credit scores), % Biden, 0.19 (0.15), and most importantly, well-being, 0.74 (0.73). The biggest difference in correlations here was for IQs predicting income inequality (−0.24 and −0.67 for NAEP and PIAAC, respectively), but I am not sure how to interpret this.

### 3.2. State Well-Being

All variables in Table 3 significantly correlated with the global well-being measure. Very strong correlations existed between global well-being and religiosity (−0.74) and FICO credit scores (0.85). In fact, of the 12 other variables in Table 3, 8 of them correlated 0.50 or more with global well-being.

It is interesting that well-being’s strongest correlations in Table 3 are with FICO credit scores (0.85), state IQ (0.80), and then religiosity (−0.74). These nicely illustrate the strongly intercorrelated nature of the g/well-being nexus. The correlation between well-being and alcohol consumption (0.66) is also notable in Table 3. As with alcohol and IQ, the correlation’s direction here is positive. That is, states that drink more have higher well-being.

### 3.3. Older State IQ Estimates

My final analysis involved first simply correlating new state IQ scores with the existing ones. State IQ derived here correlated 0.91 with McDaniel’s (2006) estimates and 0.93 with the IQ scores reported by Fuerst and Kirkegaard (2016). Current state IQ scores also correlated at an impressive 0.58 with state IQ estimates (Alexander 1922) derived more than 100 years ago. Thus, the g nexus here shows strong stability across many years.

Additionally, I briefly examined how the old (McDaniel 2006) and current IQ estimates predicted the well-being subdomains. The correlations with the old (new) IQ scores were −0.71, −0.74 for crime; 0.67, 0.74 for education; 0.42, 0.57 for health, and 0.51, 0.71 for income. Given that the old/new IQ values correlated 0.91 themselves, it is perhaps not surprising that the results with well-being are similar across the two estimates. If anything, the new IQ scores are somewhat more predictive (or the well-being variables are scaled better here) than the old values. 

## 4. Discussion

My first goal was to update McDaniel’s (2006) state IQ estimates. I did this by analyzing both PIAAC (for adults) and NAEP (for children) state-level exam scores by state. Both exams are highly g-loaded, and their combination results in IQ scores that represent state residents across the lifespan.

I tested the convergent validity of the new IQ estimates by correlating them with eleven other, important, state-level variables. IQ correlated with eight of these (nine when considering suppression effects). Specifically, values ranged from state IQ correlating −0.33 with percent gun owners to 0.87 with FICO credit scores.

A second goal was to update Pesta et al.’s (2010) well-being estimates. Here, I decided on four well-being subdomains (crime, education, health, and income), whereas Pesta et al. (2010) originally included both state IQ and religiosity as other components of well-being (I removed them from the well-being measure here due to face validity issues). I also used multiple indicators of each subdomain, and the data were coded from reliable sources such as the U.S. Federal Government, Gallup, the Pew Research Center, CNN, etc.

Four strong principal components emerged from these analyses. The components explained between 76% (crime) and 94% (income) of the variance in the well-being subdomains. The hierarchical/global PCA also produced a single well-being component, explaining 75% of the variance in the four components for the subdomains. Note also that well-being correlated 0.80 with the state IQ scores.

Next, as with IQ, I tested global well-being’s predictive power by correlating it with eleven other important variables within the well-being nexus. Well-being correlated with all these other variables. Values ranged from −0.28 (Income inequality) to 0.85 (FICO credit scores). Of these eleven correlations for global well-being in Table 3, nine are 0.50 or higher in magnitude.

What do we know from 16 years of state IQ research? First, state-level variables in general display a “positive manifold” of correlations, much like different cognitive tests do at the individual level. There is also something akin to an “indifference of the indicator” effect here for IQ and well-being. IQ, for example, seems to be reliably measured by either the adult-focused PIAAC or the children-focused NAEP exams. Likewise, many of the variables I used to scale the well-being subdomains were different from those used by Pesta et al. (2010), yet both sets of variables strongly predict other variables.

Second, state IQ emerges as a potent predictor of an impressive array of other state-level variables, as featured here and elsewhere. Third, as with individual IQ scores, the state-level IQ nexus extends beyond cognitive variables and into well-being subdomains such as crime, education, health, and income.

Fourth, state-level aggregation produces large effect sizes. For example, the individual-level correlation between income and IQ is around 0.30 (Jensen 1998). Here, it is 0.71. Likewise, the individual-level correlation between crime and IQ is around −0.20 (see, e.g., Moffitt et al. 1981). Here, it is −0.74.

Fifth, large suppression effects (see, e.g., Tzelgov and Henik 1991) sometimes exist with state IQ data. Here, the percent of state residents voting for Joe Biden failed to correlate with state IQ until I controlled for race/ethnicity (i.e., percent White). Likewise, when controlling for IQ, the negative correlation between percent White and percent Biden doubled.

## 5. Conclusions

What is perhaps most interesting about state-level data is that it is difficult to find two state-level variables that are uncorrelated, even when the relationship itself lacks face validity. As mentioned above, there is something akin to an “indifference of the indicator” effect here for state IQ together with well-being (and its sub-domains). Just some examples of strong, face invalid correlations in Table 3 include IQ and alcohol consumption (0.57), state FICO scores (0.87), and income inequality (−0.51). Other examples include global well-being and religiosity (−0.74), temperature (−0.50), and percent Biden (0.60). 

In fact, the effects here are also akin to a “well-being positive manifold,” such that swapping out “additional measures” of one construct (e.g., household income versus home values) with additional other measures of the construct is certainly reasonable. Here, however, I picked all variables based mostly on what the literature has focused on (plus personal interest). 

Additionally, one benefit to archival data is that they are widely available, and anyone can add any other aggregate-level variables to the mix to test whatever hypotheses one is interested in testing. At any rate, “nexus” seems to be the best term to describe all these various state-level relationships. In fact, that everything is strongly intercorrelated here seems rather interesting, versus something to be concerned about.

The biggest limitation to the present study is the temptation to commit the ecological fallacy (Robinson 1950). It is invalid to conclude that because something exists in the aggregate, it must also apply to individuals as well. A related potential limitation is Simpson’s paradox (Simpson 1951), wherein aggregating data sometimes leads to vastly different results than those found without aggregation. However, regarding both the ecological fallacy and Simpson’s paradox here, the variables that IQ predicts among individuals (e.g., crime, education, health, and income) are also predicted at the aggregate/U.S. state level.

Updated IQ and well-being scores might also be useful as one guide in aiding public policy decisions. For example, a state’s standing on crime, education, health, income and—perhaps especially—global well-being would give any U.S. state an additional sense of its position on these variables relative to other states. Obviously, then, resources might be directed toward the most deficient subdomains.

In sum, at the level of the U.S. state, a nexus of intercorrelated variables exists. IQ resides near the center of the nexus, albeit the IQ correlations here extend beyond merely different measures of cognition, and into the realm of well-being and its subdomains. Likewise, well-being itself is an important other predictor of state-level outcomes. The predictive power of both state IQ and state well-being is impressive.

## Figures and Tables

**Table 1:** Correlation matrix and principal components analysis for the well-being sub-domains plus state IQ.

Variable	% Variance in 1st Principal Component	Correlation Matrix					
		1	2	3	4	5	6
1 Crime	76%	--	−0.73	−0.39	−0.57	−0.76	−0.74
2 Education	92%		--	0.64	0.80	0.92	0.74
3 Health	84%			--	0.87	0.84	0.57
4 Income	94%				--	0.94	0.71
5 Global Well-being ^1^	75%					--	0.80
6 State IQ	--						--

^1^ Notes. Global well-being resulted from a hierarchical PCA of the four subdomains above it. Notice that crime loads negatively on global well-being. Note also that state IQ was derived separately from the well-being variables.

**Table 2:** State ranks and standard scores for IQ and the well-being measures.

State	IQ Rank/Score	Well-Being Rank/Score	Crime Rank/Score	Education Rank/Score	Health Rank/Score	Income Rank/Score
Alabama	47/96.4	45/76	47/80	44/81	46/77	44/79
Alaska	29/99.4	24/103	43/80	28/98	3/120	13/108
Arizona	37/98.3	39/91	39/89	42/84	27/102	35/93
Arkansas	44/97.1	48/71	49/75	47/79	49/70	46/76
California	45/97.1	17/108	37/90	29/97	4/118	8/119
Colorado	13/101.1	9/116	40/89	12/112	1/127	4/121
Connecticut	11/101.2	3/122	4/122	2/128	10/112	11/113
Delaware	35/98.7	28/100	29/96	14/109	41/89	23/103
Florida	34/98.8	36/92	38/90	34/93	31/98	37/92
Georgia	40/98.1	34/93	36/90	33/93	32/98	34/94
Hawaii	32/99.2	10/115	23/101	19/106	5/117	1/126
Idaho	20/100.5	25/103	9/117	38/88	18/107	26/101
Illinois	31/99.4	19/106	28/97	11/114	17/107	22/103
Indiana	19/100.6	32/94	19/104	36/91	36/91	33/95
Iowa	14/101.1	20/106	10/116	23/101	21/106	24/101
Kansas	21/100.5	30/97	35/90	25/100	30/100	28/100
Kentucky	33/98.8	41/83	15/111	41/85	48/71	45/79
Louisiana	49/95.2	46/74	50/73	45/80	43/84	48/74
Maine	17/100.9	22/106	1/128	13/111	42/88	29/98
Maryland	25/100	8/116	27/100	5/122	14/109	3/122
Massachusetts	2/103.1	1/126	8/117	1/131	7/116	2/125
Michigan	26/99.6	31/94	25/101	27/98	38/91	39/91
Minnesota	3/102.9	5/119	12/114	9/116	6/116	9/117
Mississippi	48/95.8	49/71	32/92	50/74	47/73	50/66
Missouri	27/99.5	40/89	42/81	30/97	37/91	38/92
Montana	15/101.1	27/101	33/92	16/107	16/109	32/95
Nebraska	10/101.2	21/106	24/101	18/106	19/107	16/107
Nevada	46/96.6	38/91	34/91	48/79	29/100	27/101
New Hampshire	1/103.2	4/121	2/126	6/121	20/106	7/120
New Jersey	16/101.0	2/124	5/121	3/128	11/112	6/121
New Mexico	50/95.0	43/80	44/80	43/81	33/97	47/74
New York	36/98.4	13/112	16/108	7/118	15/109	17/105
North Carolina	28/99.5	35/93	30/96	31/95	35/94	40/90
North Dakota	5/101.7	16/108	17/108	26/100	8/112	15/108
Ohio	24/100.0	37/92	26/100	35/92	39/90	36/92
Oklahoma	39/98.2	44/79	45/80	46/80	44/83	43/83
Oregon	22/100.3	23/104	21/103	22/101	25/104	18/105
Pennsylvania	23/100.2	26/102	18/106	17/107	34/96	30/97
Rhode Island	30/99.4	12/112	7/118	10/115	28/102	14/108
South Carolina	41/97.8	42/83	48/77	40/86	40/89	42/87
South Dakota	18/100.7	29/98	31/96	32/93	26/104	25/101
Tennessee	38/98.3	47/72	46/80	39/88	45/81	41/89
Texas	42/97.4	33/93	41/84	37/89	23/106	31/97
Utah	8/101.5	6/118	13/114	20/103	2/124	5/121
Vermont	4/102.2	7/117	3/124	4/126	24/105	19/105
Virginia	9/101.2	11/114	11/116	8/116	22/106	12/112
Washington	7/101.5	14/111	22/102	15/109	12/110	10/116
West Virginia	43/97.2	50/71	20/103	49/77	50/54	49/71
Wisconsin	12/101.2	18/108	14/111	21/103	13/109	21/105
Wyoming	6/101.7	15/110	6/120	24/100	9/112	20/105

*Notes:* All variables were converted to *M* = 100, *SD* = 15. For state-level IQ, though, the scores were derived first from the standard deviations of the individual PIAAC and NAEP exam administrations.

**Table 3:** A well-being nexus of correlations, including IQ, global well-being, and various state-level measures of interest.

Variable	1	2	3	4	5	6	7	8	9	10	11	12	13
1. IQ	--												
2. Well-being	**0.80**	--											
3. Religiosity	**−0.57**	**−0.74**	--										
4. Income inequality	**−0.51**	**−0.28**	**0.28**	--									
5. Conservatism	**−0.34**	**−0.68**	**0.71**	−0.04	--								
6. COVID vaccinated	**0.38**	**0.68**	**−0.66**	0.10	**−0.83**	--							
7. Price of cigarettes	0.19	**0.49**	**−0.59**	0.10	**−0.66**	**0.58**	--						
8. Alcohol consumption	**0.57**	**0.66**	**−0.73**	−0.16	**−0.61**	**0.59**	**0.37**	--					
9. Biden (%)	0.18	**0.60**	**−0.60**	0.20	**−0.87**	**0.85**	**0.63**	**0.52**	--				
10. Minimum wage	0.13	**0.41**	**−0.57**	−0.19	**−0.71**	**0.67**	**0.57**	**0.36**	**0.66**	--			
11. Temperature	**−0.62**	**−0.50**	**0.60**	**0.54**	0.22	−0.21	**−0.27**	**−0.48**	0.00	−0.19	--		
12. Gunowners (%)	**−0.33**	**−0.51**	**0.38**	−0.16	**0.52**	**−0.55**	**0.26**	**−0.39**	**−0.53**	**−0.33**	0.06	--	
13. FICO credit score	**0.87**	**0.85**	**−0.74**	**−0.46**	**−0.53**	**0.51**	**0.36**	**0.70**	**0.35**	**0.36**	**−0.66**	**−0.34**	--

Note. A correlation of *r* = 0.24 is significant at *p* < 0.05, as shown in bold.

## Data Availability

Not applicable.
